# Safe *Hb* Concentration Measurement during Bladder Irrigation Using Artificial Intelligence

**DOI:** 10.3390/s21175723

**Published:** 2021-08-25

**Authors:** Gerd Reis, Xiaoying Tan, Lea Kraft, Mehmet Yilmaz, Dominik Stephan Schoeb, Arkadiusz Miernik

**Affiliations:** 1 Department Augmented Vision, German Research Center for Artificial Intelligence, 67663 Kaiserslautern, Germany; xiaoying.tan@dfki.de; 2 Medical Centre, Department of Urology, Faculty of Medicine, University of Freiburg, 79106 Freiburg, Germany; lea.kraft@uniklinik-freiburg.de (L.K.); mehmet.yilmaz@uniklinik-freiburg.de (M.Y.); dominik.stefan.schoeb@uniklinik-freiburg.de (D.S.S.); arkadiusz.miernik@uniklinik-freiburg.de (A.M.)

**Keywords:** hemoglobin sensor, bladder irrigation monitor, absorption near infrared, artificial intelligence, bubble detection

## Abstract

We have developed a sensor for monitoring the hemoglobin (Hb) concentration in the effluent of a continuous bladder irrigation. The Hb concentration measurement is based on light absorption within a fixed measuring distance. The light frequency used is selected so that both arterial and venous Hb are equally detected. The sensor allows the measurement of the Hb concentration up to a maximum value of 3.2 g/dL (equivalent to ≈20% blood concentration). Since bubble formation in the outflow tract cannot be avoided with current irrigation systems, a neural network is implemented that can robustly detect air bubbles within the measurement section. The network considers both optical and temporal features and is able to effectively safeguard the measurement process. The sensor supports the use of different irrigants (salt and electrolyte-free solutions) as well as measurement through glass shielding. The sensor can be used in a non-invasive way with current irrigation systems. The sensor is positively tested in a clinical study.

## 1. Introduction

In urology, continuous bladder irrigation (CBI) is an important standard care procedure [1,2,3,4] after transurethral resection of the bladder (TURB) or the prostate (TURP). The dominant goal of CBI is to prevent the formation of blood clots and consecutive bladder tamponade, a medical condition requiring an additional and foremost avoidable follow-up surgery [5]. The purpose of CBI in the given application scenario is, therefore, to keep the blood concentration in the bladder at a very low level. Technically, CBI provides a continuous dilution of the bladder content with fresh irrigation fluid (often saline) and thus, prevents clot formation. Although nearly trivial from a pure technical point of view, CBI is involved when applied in clinical practice. Improper CBI may trigger bladder spasms by irritating the bladder, cause undesired bleeding [5], lead to bladder rupture or perforation [6], and might even become life-threatening [7]. These and other possible complications result primarily from increased pressure, due to a high flow rate. Consequently, there are two optimization goals for an optimally adjusted CBI: On the one hand, the flow should be high enough to dilute the blood sufficiently, and on the other hand, the flow should be as low as possible to avoid pressure-related complications. Since the amount of bleeding after surgery cannot be controlled, the optimal flow speed changes over time. Hence, CBI demands extensive and continuous supervision and management by medical personnel, imposing a heavy burden on nurses responsible for a whole urological station [8,9].

CBI supervision is, on the one hand, comprised of rather technical aspects, such as caring for a filled fluid reservoir and an empty waste reservoir, ensuring a continuous flow of fluids into and out of the bladder. On the other hand, there are aspects with high medical relevance, in particular, choosing the right irrigation flow. Too high a flow will keep the blood concentration low, but may cause severe negative effects to the patient. In the case of the urethral catheter becoming clogged by a clot, continued irrigation may even lead to bladder rupture [5]. Too low a flow will not suffice to dilute the blood and will result in an ineffective procedure.

Today, nurses inspect the coloring of the waste fluid and estimate the flow speed accordingly. Obviously, this approach has significant drawbacks. Estimating the blood concentration in the outflow demands a high level of experience and is observer-dependent. Furthermore, it is affected by external conditions, such as the current illumination. Additionally, nutrition can have a significant effect on urine color, such as betanins leading to beeturia [10], easily confused with hematuria. Other foods known to change urine color include blackberries and rhubarb, which turn urine pink or red, while fava beans and aloe turn it reddish brown. Medications can also affect the color of urine, such as phenazopyridine, a drug used to numb urinary symptoms, the antibiotic rifampin, and laxatives containing senna, which can turn urine reddish-orange. The anti-inflammatory drugs sulfasalazine and phenazopyridine, as well as certain chemotherapy drugs turn urine orange-red, while the antimalarials chloroquine and primaquine, the antibiotics metronidazole and nitrofurantoin, the muscle relaxant methocarbamol, and laxatives containing cascara turn urine reddish-brown. In addition to the drug itself, the food coloring contained in the coating, e.g., in the case of tablets, pills, and dragees, can also discolor the urine. The discoloration of the urine and, thus, of the waste fluid is easily mistaken for an increased amount of blood, which in turn leads to an increased irrigation flow. The color of the excretory fluid is, therefore, not a reliable indicator for the adjustment of the flow. Detection of acute bleeding might become obscured, again putting patients at risk.

In order to better estimate the blood concentration, Hageman et al. [11] developed the Hemostick, a color scale to visually compare blood color. While this device significantly contributes to standardize the estimation of blood concentration between nurses, it still is affected by illumination influences. Furthermore only a limited number of discrete reference colors are available. Therefore, as can be observed in Figure 9, the distinction of blood concentration based on observation or color comparison becomes quickly unfeasible. Ding et al. [12] evaluated a CBI control system able to adjust the irrigation flow automatically based on the estimation of the blood concentration. The system features a color monitor to estimate the blood concentration. It thus resembles an automated version of the approach presented in [11]. In [13], Chan et al. presented a device to measure the blood concentration, using the light absorption principle. In order to estimate the blood concentration, they investigated light emitting diodes (LED) with different colors (i.e., red, green, blue) and finally decided on using green LED. Unfortunately, they did not specify the particular wavelengths of the used LEDs. To measure the transmitted light, a light dependent resistor (LDR) was used. Timm et al. [14] proposed a system based on light absorption for non-invasive estimation of hemoglobin (Hb) concentration in human tissue. They used three different, well-defined wavelengths to estimate the Hb concentration. The system was tested in a technical setting. Zhang et al. [15] described a system to estimate blood loss during endoscopic surgery based on Hb measurements. Their system is also based on absorption since they use a photoelectric sensor. Unfortunately, they did not provide any specifics on the sensor.

The new Hb concentration sensor proposed in this article also exploits the light absorption principle and thus, is closely related to the work in [13,14,15]. The advantage of using the absorption principle compared to color monitoring is the improved accuracy of the estimates. Independence from dietary coloring effects can be achieved through the selection of appropriate light frequencies. In contrast to [13], our approach is far more rigorous. The light absorption properties of blood and other coloring components were taken into account, and an optimal light frequency was selected. In contrast to [14], our system is dedicated to monitor CBI. The measurements take the light absorption of the irrigation tubing into account, as well as influences of the irrigation fluid. In contrast to [15], our system measures the Hb concentration in a defined tube in contrast to being integrated in a collecting bucket. In addition, practical aspects, such as gross mismeasurements due to bubble formation in the outflow tubes and measurement through thin glass panes, were taken into account. The system was evaluated in a clinical study.

The proposed sensor is part of a comprehensive mobile CBI monitoring system. Although the overall system is outside the scope of this paper, the main reasons for its developments will be briefly presented. As mentioned earlier, continuous monitoring of CBI is important. In this regard, a single nurse can take care of about two to four patients, provided that they are in the same room and the nurse has no other duties. In reality, however, there are easily twenty concurrent CBI on a urology ward, spread across multiple rooms and cared for by a single nurse. Therefore, continuous monitoring of all patients is not feasible. In addition, the nurse must maintain the CBIs by emptying or changing the waste bags and replacing empty saline bags in a timely manner. Finally, the nurse also has to manage the daily routine of attending to the well-being of patients, dispensing medications, conferring with physicians, and much more. Regardless of what a nurse is doing, he/she always has the pressure in the background that he/she should be monitoring CBIs. This has several negative consequences. Nurses are under constant stress, even when monitoring a particular CBI because other CBIs cannot be monitored at the same time. Nevertheless, acute bleeding in one or more patients can occur at any time. On the other hand, maintenance of the CBIs also places a burden on the nursing staff since overflowing waste bags as well as empty reservoir bags should be avoided as much as possible. The monitoring system relieves the nursing staff of this pressure since the flow rate and Hb/blood concentration are constantly monitored by a technical system. The flow and concentration measurements can be used to effectively calculate whether irrigation is being performed optimally. As soon as one of the parameters leaves its predefined limits, alarms can be transmitted to a mobile device and/or to the ward room. In addition, the current status of all CBIs can be visualized simultaneously, giving the nurse an optimal overview of all patients. The use of the monitoring system allows nurses to fully concentrate on their respective tasks, while giving them the security of knowing that they will be informed in time if intervention is required. Overall, the system thus provides the opportunity to improve CBI monitoring and thus patient care, while at the same time reducing the workload of nurses, thus indirectly further improving patient care. The proposed sensor is an extremely important and integral component of such a monitoring system. Additional fields of application are conceivable, but will not be considered further at this point.

## 2. Materials and Methods

### 2.1. Selecting the Measurement Method

A requirement for the sensor development was the seamless integration with existing CBI systems. A refractometer-based approach or the use of other sensors that require direct contact with the medium to measure Hb concentration is, therefore, inappropriate. The Hb measurement in this work is performed based on light absorption. A known amount of light is exerted by a LED, passed through a tube filled with CBI waste fluid and captured on the opposite side. According to Beer’s Law, the concentration is then related to the intensities and measurement section via the following:

$$I_{t}=I_{0}\cdot 10^{-\varepsilon \Delta zc},$$

where $I_{t}$ and $I_{0}$ denote the transmitted and incident light intensities, respectively, ε and *c* denote the molar absorptivity and concentration of the light absorptive compound, and Δz is the optical path length through the drainage fluid. By selecting a proper power supply and wavelength for the LED, we obtain constant $I_{0}$ and ε. When the outflow tube is full of drainage fluid, Δz is also constant and determined by the diameter of the tube. Therefore, we can infer the Hb concentration *c* solely based on $I_{t}$.

### 2.2. Selecting the Light Source

In this simple form, Beer’s Law is only valid for a single light absorbing material. The waste fluid is, however, composed of various materials, i.e., irrigation fluid and potentially additional medications, as well as urine and blood. The latter two materials are compounds on their own. We, therefore, investigated how strong the influence of the additional ingredients would be. In a work from Pegau et al. [16], we found that the influence of salt on the absorption property of water is very small in the near infrared frequency band and that salt might even lower absorptivity. From Palmer et al. [17], we learned that the absorption of water compared to Hb is negligible (approx. ratio 1:40,000). Additionally, urine coloring molecules, such as betanins, have their main absorption in the range of 400–600 nm and absorb only very little light in the near infrared as Gonçalves et al. [18] reported. In summary, we can state that Hb absorption is absolutely dominant, particularly in the near infrared but also in the visible spectral band. Hence, we apply Beer’s Law in its simple form without danger of significantly degrading the measurement accuracy.

**Figure 1:**
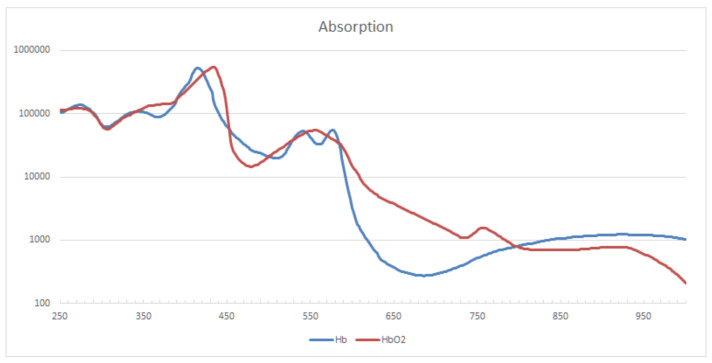
Molar extinction coefficient e in $\left[\frac{L}{\mathrm{mol}\cdot \mathrm{cm}}\right]$ of Hb and $HbO_{2}$ for wavelengths between 250 and 1000 nm. Major isobestic points are located at 420, 545, 570 and 800 nm.

The absorption curve of Hb and $HbO_{2}$ is shown in Figure 1. For the formation of blood clots, it is basically irrelevant whether Hb is oxygenated or not. In order to account for both Hb versions at the same time, isobestic points, i.e., points at which two chemical species have the same molar absorptivity, should be used for measurement. For Hb and $HbO_{2}$ the main isobestic points are at frequencies of 420, 545, 570, and 800 nm. In tests with blood samples and the targeted tubing, it was found that the absorption for the lower frequency points is way too high for a CBI monitoring system. The transmitted amount of light quickly drops below values that can be reliably distinguished from background noise. Additionally, total absorption is reached very quickly, so increasing the light intensity would not help. Combined with knowledge of the absorption properties of other relevant materials, these findings led to the determination of the central measurement frequency at 800 nm.

**Figure 2:**
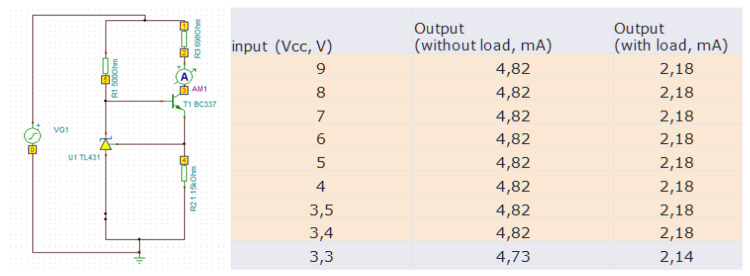
LED needs a constant current to emit a well-defined light. (**Left**): Circuit diagram of the used electronic. (**Right**): Test results with and without load. Constant current can be guaranteed for a wide range of input voltages.

For an LED, the emitted light intensity as well as the light frequency depend on the applied current. Especially when using batteries in a mobile setting, care must be taken to provide a constant current supply. To this end, we implemented a small electronic component (see Figure 2), that guarantees a constant current over a wide voltage range. Implementation was done on a stripboard, using regular size electronic components.

### 2.3. Selecting the Light Sensor

In order to measure the transmitted light, a photo resistor (LDR), or a photo diode (PD) would theoretically be sufficient, as shown in [13]. Taking practical considerations into account, i.e., the fact that the CBI tubes do have reinforcing ribs, a single measurement is not sufficient for a reliable monitoring system. Even more problematic is the fact that bubbles are traveling unpredictably in the outflow tube. Gain ribs appear darker in the detector image and suggest increased Hb values, while the presence of bubbles dramatically decreases light absorption and suggests lower Hb values. While slightly increased Hb readings might be tolerable, significantly lower readings would put patients at risk. As a consequence, measuring the Hb concentration with a single point measurement (LDR/PD) is very dangerous in practice. We propose to use a camera chip instead. At only moderately increased cost, a wealth of measurements can be performed, enabling the application of advanced image processing techniques.

For system demonstration, we chose a monochrome camera without an IR filter, which provides us with 10 bit linear intensities. While the concrete camera model is irrelevant, the three mentioned properties of the camera are important. A monochrome camera lacks a Bayer filter; hence, all pixels generate readings of the same quality. The lack of IR filtering allows for reliable measurements in the near infrared frequency band. The 10 bit color depth increases the dynamic range and provides an increased measurement range.

### 2.4. Extending the Measurement Interval

Considering the exponential relationship between the intensity and Hb concentration, the dynamic range of even a 10 bit sensor is rather limited. Starting from an exposure $E_{Low}$ that just saturates the sensor for pure irrigation fluid, the maximum measurable Hb concentration is less than 0.6 g/dL (equivalent to ≈4% blood). Increasing the exposure time by a factor of 8 ($E_{High}$) allows measurements of more than 3.2 mg/dL Hb (≈20% blood), but leads to overexposure for the Hb concentration below 0.1 g/dL. We, therefore, introduced a dual-exposure setup, which is controlled, using a hysteresis threshold $T_{Low,High}$. We chose $T_{Low}$ (i.e., where we switch from $E_{High}$ to $E_{Low}$) at 0.2 g/dL Hb and $T_{High}$ at 0.6 g/dL Hb. In terms of sensor readings, i.e., intensities, the $T_{Low}$ is at 800 and the $T_{High}$ is at 80. The overall measurement range of the proposed sensor, therefore, covers the interval [0–3.23] g/dL hemoglobin concentration or [0–21.5]% blood concentration.

### 2.5. Processing of Sensor Images

We apply three different image processing methods, namely the detection of shadow artifacts caused by the gain ribs, detection of bubbles, and automatic exposure adjustment.

**Figure 3:**
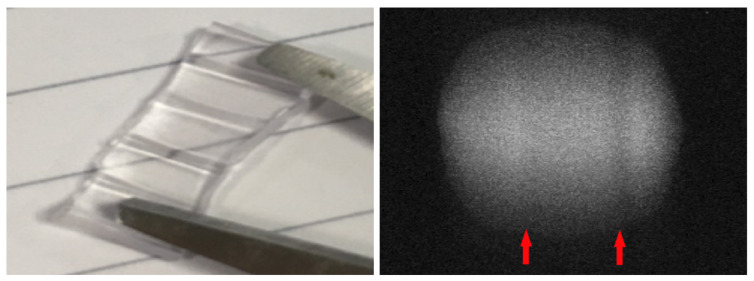
(**Left**): A segment of the waste fluid tube. Reinforcement ribs are running along the tube. The ribs significantly change the optical pass of light traveling through the tube. (**Right**): Acquired sensor image with marked artifacts caused by reinforcements. The horizontal location of the shadows depends on the tube placement and cannot be controlled. We note that the right figure was intensity adjusted for better depiction, which amplified intensity variations in the vertical direction as well as the noise level.

**Figure 4:**
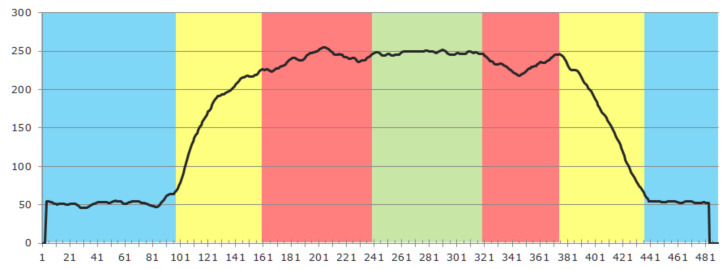
Exemplary intensity profile segmented into regions: (blue) background region, (yellow) transitional region, (red) artifact region, (green) measurement region.

Figure 3 gives an impression of the effect created by the gain ribs. The left subfigure shows a small, opened segment of the tube placed on a white paper with parallel pencil lines. Significant distortion of the lines can be observed near the ribs, which act like plano-convex lenses. The subfigure on the right shows the effect of the ribs on the detector image. The horizontal position of the artifacts depends on how the tube was inserted into the sensor. Vertical line artifacts with mainly decreased intensity can be observed. In some cases, even a slight intensity increase can occur, depending on the positioning of a rib within the optical pass. Although the positioning of the tube cannot be controlled in practice, the artifacts can be reliably detected. Since the tube does not change its position during the entire CBI process, it is practical to automatically select an artifact-free measurement ROI when setting up the system. This can be realized by inspecting the intensity profile of a horizontal line and comparing it to a known intensity profile. An exemplary profile is depicted in Figure 4. The blue regions are those that are not directly illuminated, while the yellow regions are transitional zones that are partially lit. Both regions are a constant size and determined during the sensor assembly since they solely depend on the actual camera and LED placement. The union of the red and green regions is the potential measurement area. In the example, we detected two artifact regions, marked in red, and an artifact-free region, where we can safely place the measurement ROI.

**Figure 5:**
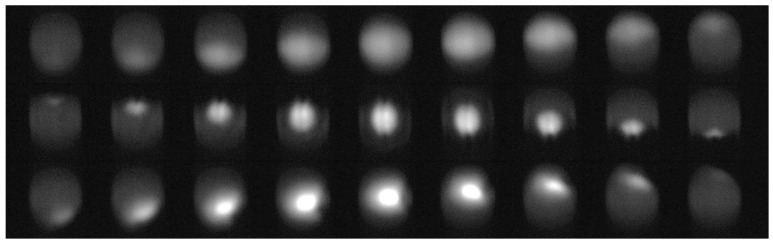
Examples of the appearance of bubbles in the sensor image. (**Upper row**): bubble passing the sensor close to the LED. (**Middle row**): bubble passing the sensor close to the camera. (**Lower row**): bubble passing near the center of the tube. For all images, the same specimen was used, so Hb concentration as well as bubble size are the same.

**Figure 6:**
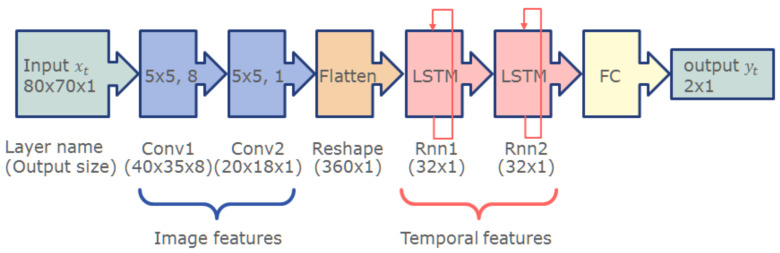
Network to detect bubbles in the optical path. The convolutional layers extract image features, such as bright and dark edges, bright spots, etc., while the LSTM layers account for the temporal components. The network is able to detect bubbles traversing in both directions and even accounts for those that remain static in the optical path for an extended time.

As already mentioned earlier, bubbles pose a significant problem to the measurement process. While the gain ribs create static artifacts that only slightly and systematically alter the measurements, bubbles are dynamic by nature and severely impact the measurement. Figure 5 demonstrates the effect of a bubble in the sensor image. Please note that for all image sequences, the same specimen was used, i.e., a small tube section filled with diluted blood and sealed with a small fraction of air, similar to those depicted in Figure 9. Striking is the difference in the image sequences depicted in Figure 5. The only difference between the sequences is the location with respect to the camera and LED, where the bubble travels along the tube. Already, these examples demonstrate that a bubble detection based on classic approaches is not feasible. The situation becomes even more challenging when several small bubbles form a foam-like cluster. Furthermore, we found that also pure image processing of a single image is not sufficient to reliably detect bubbles: in the case that a bubble of a reasonable size remains static within the light pass, the images can be indistinguishable from images without bubbles at a lower Hb concentration. It is, therefore, imperative to use a system that can analyze image sequences. To this end, we trained a convolutional recurrent neural network comprised of convolution layers to extract optical features followed by long short-term memory (LSTM) layers to account for the temporal aspect. The network was trained on video sequences acquired with the sensor hardware. To this end, test specimen with various blood concentrations and bubble sizes were prepared (see Section 2.8). The network was trained fully supervised. The ground truth was generated by manually marking the images in the sequences where a bubble just entered the view or almost completely left the view. The remaining images were classified automatically. Figure 6 depicts the network architecture. For details on the network and how it was trained, we refer the interested reader to [19].

### 2.6. Sensor Signal Processing

Sensor signal processing, i.e., bubble detection, Hb value generation, and communication, was implemented on a Raspberry Pi 4B. The camera was connected via the USB 3 interface, which also provided power to the LED. Furthermore, the Raspberry Pi was used to communicate the data to the main system and to control additional sensors that are out of the scope of this paper. The operating system used was a completely stripped-down Ubuntu Linux with a custom compiled kernel.

The bubble detection network was run on the ARM-CPU exploiting the ONNX Runtime [20]. The camera provides images at a frame rate of 25 fps. All images are analyzed by the network. Sequences of 8 images are combined to guarantee stable outputs, even in the case that the network fails to detect a bubble or falsely detects a bubble in an image. The output frequency of the sensor was set to 10 measurements per second. Data were provided to the user side (C# DLL) by means of a double buffer. Overall, power consumption for the running system is below 700 mA.

**Figure 7:**
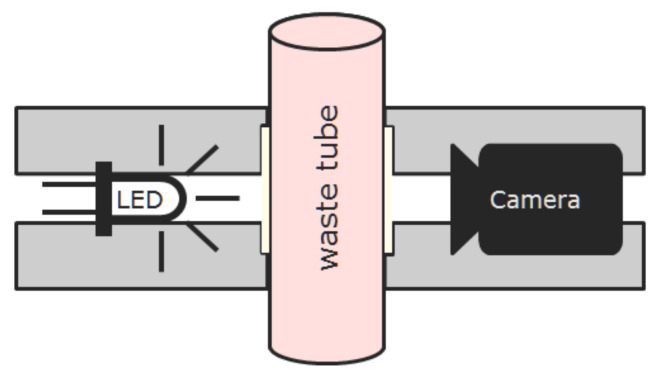
Schematic representation of the sensor. On the left side, an LED exerts light that is transmitted through the tube filled with irrigation waste fluid. The transmitted light is captured by a camera. The bright squares next to the tube represent thin glass plates that can be inserted into the optical pass to seal the LED and the camera and to enable effective cleaning and disinfection in clinical application.

To further support future integration of the sensor, we quantized the network to fixed point representation (Figure 7), using a quantization aware training (QAT) method. The resulting network was implemented on FPGA (Xilinx xc7z020-clg400-1). As shown in Table 1, the standard version of the network still leaves sufficient resources for additional hardware, such as a MIPI camera interface as well as the calibration tables to convert sensor readings into Hb values. Thus, the whole signal processing can be performed on a single SoC of a size less than 20 × 20 × 2 mm. This not only reduces the overall sensor size, but also significantly lowers the overall power consumption and, in turn, enables mobile CBI monitoring.

### 2.7. Sensor Housing

A sensor used in clinical practice needs to be cleaned and disinfected. Neither the camera lens nor the LED are well suited for this process. Besides potential deterioration effects, camera lenses feature sharp edges where bacteria and other germs might survive. It is therefore mandatory to seal the optical components. A particularly suited material for the case at hand appears to be glass. We conducted a series of tests using glass cover slips for microscopic samples with a thickness of 0.15 mm ± 0.02 mm. Assuming circular openings for the camera lens (∅ ≈ 1.0 cm) and the LED light channel (∅ ≈ 0.5 cm), the cover slips appear to be sufficiently robust.

Since the positions of the glass plates, camera, and LED are rigid and only light frequencies in a very small band are used, we confirmed in tests that the influence of glass is a constant offset to the measurement. This was expected by altering Beer’s Law in the following way:

$$I_{t}-\Delta I_{t}=\left(I_{0}-\Delta I_{0}\right)\cdot 10^{-\varepsilon \Delta zc},$$

where $\Delta I_{t}$ and $\Delta I_{0}$ denote the attenuation of the exerted and captured intensity by the two glass slips.

The housing itself was 3D printed from black acrylonitrile butadiene styrene (ABS). Tests with white ABS showed significant light penetration in the sensor, which greatly affected the measurements. Although it would be possible to shield the measurement path by other means, using black material seemed to be the most direct approach.

**Figure 8:**
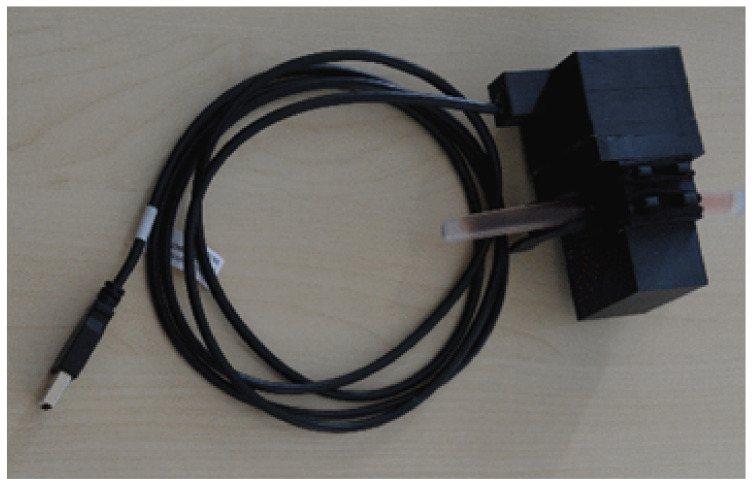
Image of the assembled sensor as used in the clinical validation study. Communication and power supply were realized using a USB 3 interface. A short tube segment was inserted to showcase sensor attachment to the CBI system.

In contrast to [13], we decided for a rigid housing instead of a clamp. While a clamp has the advantage that it can be most easily attached to a variety of tubes, it is much harder to know the true length of the measurement distance. Furthermore, the clamp pressure deforms the tube and thus introduces uncertainty. Lastly, it is much harder to avoid ambient illumination to impact on the measurement. An image of the sensor is given in Figure 8. Three versions of the sensor were assembled and provided for integration.

### 2.8. Preparation of Test Specimen

**Figure 9:**
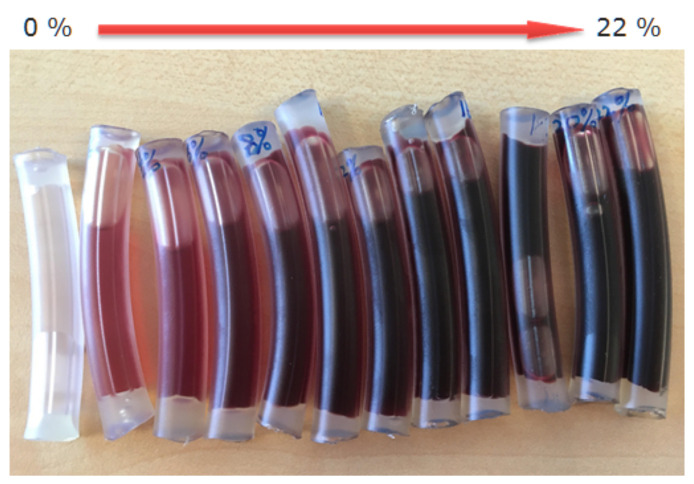
Test specimen samples. Tube segments were filled with diluted blood and sealed with hot glue. In order to test the effect of bubbles, a small fraction of air was left inside. This also prevented direct contact between the hot glue and test liquid. Samples were produced for blood concentration between 0% and 22% in increments of 2%. Hb concentration was measured using a hemoglobin analyzer.

Figure 9 depicts a set of test specimen used for sensor development. A complete set always covered a concentration range between 0% and 22% in 2% increments, i.e., 12 samples. Each specimen was manufactured in two different versions: one with saline irrigation fluid and one with an electrolyte-free irrigation fluid. The latter is mainly used during surgery, but opened bags are used up afterwards.

To produce the test specimens, the drain tube of an irrigation system was cut into pieces approximately 6 cm long. One end of the tube segments was sealed with hot glue. Percentage indicators were written on each tube, using waterproof ink. This was done in a preparatory phase to avoid any delay when working with the blood.

The blood used for development was fresh porcine blood obtained immediately after slaughter. A total of 6 mL diluted Heparin (250 IU/mL) was added to 200 mL blood to prevent clotting (coagulation), and the mixture was carefully stirred to avoid sedimentation/separation. Since the proposed sensor actually measures the Hb concentration, the ground truth Hb value was measured using a hemoglobin analyzer (Measuring range: 0–25.5 g/dL; Imprecision (within run): CV < 1%; Calibrated against HiCN reference method.). Then, the blood was diluted with the respective irrigation fluid. The prepared liquid was poured into the tube segment, leaving approximately 0.5–1.0 cm of air, and the tube was sealed with hot glue. Leaving a small air bubble in the tube served two purposes: first, the presence of an air bubble allowed the effect of air bubbles to be studied accurately during scanning. Second, the air prevented possible adverse effects from the hot glue coming in direct contact with the liquid.

Samples prepared in this way provided valid measurements for one working day. Repeated measurements the following day showed significant deterioration, even when the samples were stored in the refrigerator.

In order to assess the effect of urine, additional test specimens were prepared. Human urine, collected immediately after waking up, was used for this purpose. With this urine, we were able to ensure a maximal content of salts and other metabolites. Tests on pure urine samples and samples composed of blood, urine and irrigation fluid revealed no significant effect on the measurements.

## 3. Results

**Figure 10:**
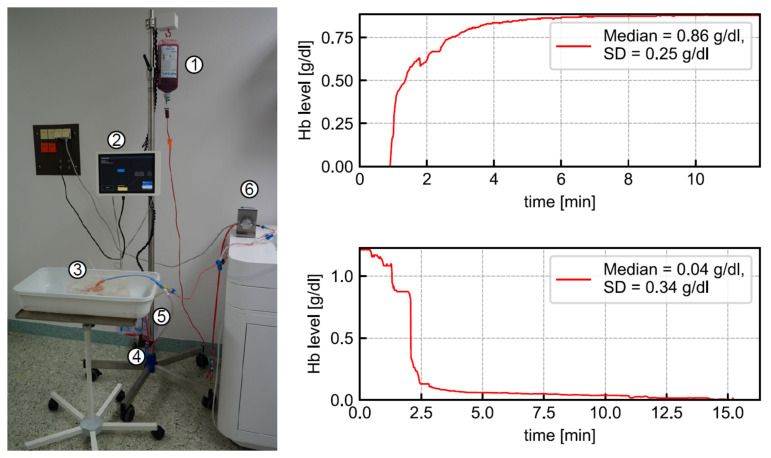
(**Left**): full system setup for clinical laboratory trials using blood from human donors: (1) blood donor, (2) display, (3) 3D bladder model, (4) Hb sensor, (5) urine bag, and (6) pump. The sensor was used in an extended setup, which is not in the scope of this paper. (**Right**): two exemplary measurement curves for increasing and decreasing Hb concentrations. Sensor time constants were estimated as 33 s for rise and 119 s for fall.

In a planned self-experiment, the sensor was tested using blood from three human donors. Using human blood donations did not require ethics approval, according to the local ethics council. The full laboratory setup of the CBI monitoring system is shown in Figure 10 (left). The liquid flowed from the upper bag via a Foley catheter through a 3D printed bladder model into the urine bag. A pump controlled the rate of inflow. The Hb sensor was mounted to the outflow tube to detect the Hb level for data collection. To obtain five different blood concentrations, we prepared five bags filled with 500 mL saline solution and varied amounts of blood. As a reference measurement, the Hb concentration of each bag was measured via blood gas analysis (BGA). The average difference of the sensor-detected Hb level from the BGA-detected Hb level was 0.29 g/dL. The Hb concentration in human blood is 15 g/dL on average, so the measurement error with regard to the full measurement interval was estimated as ~10%. We note that the full measurement range of the sensor was tested in the laboratory trials. Since measurement accuracy drops significantly at higher concentrations, an increased mean deviation from the BGA was to be expected. Table 2 gives a rough estimate of the measurement accuracy for some blood concentrations. The highest blood concentration that can be quantified by the sensor is roughly 20%, i.e., 3 g/dL Hb concentration.

Additionally, we employed random Hb concentrations to model the rise and fall in Hb level to simulate a real-life complication scenario, presented as a graph of the Hb level over time as depicted in Figure 10 (right). To imitate acute bleeding, blood was injected into the 3D-printed bladder model. The blood supply was replaced with a saline solution to resemble stopped bleeding. In the experiments, the sensor time constants for the CBI system, with the sensor placed near the waste bag, were 33 s for rise and 119 s for fall. Accordingly, the sensor’s response times were 2.7 min for rise and 9.9 min for fall. However, we would like to point out that these values strongly depend on temperature, irrigation flow, emissivity of the material and size, i.e., length and diameter of the respective irrigation tube, including the catheter. Furthermore, bubble detection was tested. The sensor reliably recognized random air bubbles or artificially generated air bubbles in the outflow tube.

**Figure 11:**
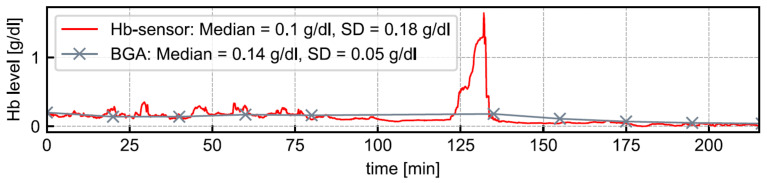
Measurement curve for a real patient. The blood gas analysis agrees well with the sensor readings. At approximately minute 125, the irrigation was inadvertently left off after the urine bag was emptied, resulting in a real-life example of the importance of the continuous monitoring of CBI.

After receiving a positive ethical vote, a clinical trial involving 20 patients was conducted to test the sensor and other components of the CBI monitoring system. All patients were male (mean age: 73.1 years) and underwent TURB. CBI was administered as part of the standard treatment procedure. Further patient information was not collected to keep the data protection footprint low. For each patient, Hb levels were monitored for at least three hours, using the CBI monitoring system. As a reference measurement, we regularly checked the Hb level in the urine bag via BGA. We would like to point out that, unlike the Hb sensor, our BGA device did not allow to measure the Hb level continuously. The Hb level detected by BGA is the average of several minutes. Figure 11 depicts an example of the sensor-detected Hb level’s development illustrated as a red line. As gray data points, ten BGA-detected Hb levels are presented. The median Hb values are at a comparable level. The mean deviation of the sensor-detected Hb level from that of the BGA-detected Hb level across all patients was −0.003 g/dL. Between minute 120 and 140, we noted a rise in Hb levels. The reason was an incorrectly configured inflow speed of saline solution. As the inflow speed was accelerated, the Hb level decreased to normal. Thus the sensor reliably measured Hb levels within a clinically acceptable deviation and with a high responsiveness across all experiments. In the patient study, the functionality of the Hb sensor’s air bubble detection could also be positively confirmed.

## 4. Discussion

As shown in the results, we were able to design a sensor to measure the Hb concentration in the effluent of a CBI with decent accuracy (±0.003 g/dL). While accuracy is highest for very small concentrations, it drops for higher concentrations, due to the exponential relation between concentration and absorption. However, for practical application this does not have negative implications. Already at 10% blood concentration, medical personnel should consider intervention. In case of a low irrigation flow, the flow should be increased to prevent cloth formation. In case of an already high irrigation flow, acute bleeding should be assumed. To increase measurement accuracy, we extended the measurement interval, using multiple exposure times. Further extension using even higher exposure times does not improve the situation, since total absorption occurs—given the fixed tube diameter.

We decided on a rigid sensor housing to achieve the most reliable measurements for system demonstration. This, of course, comes at the drawback that only a particular tube diameter is supported by the sensor. However, insets can be easily designed to account for various tube diameters. Vastly larger tube diameters decrease the measurement interval significantly since total absorption occurs earlier, while smaller tube diameters increase the measurement interval. In any case, an individual re-calibration of the sensor will be necessary.

The effect of salt on the measurement cannot be completely ignored. As can be found in the work of Pegau et al. [16], salt lowers the absorption of near infrared light by water and thus, can lead to an underestimation of the Hb concentration. As a consequence, it is definitely necessary to calibrate the sensor for the respective irrigation fluid. However, the amount of salt introduced by urine is negligible and does not have a significant effect on the measurement accuracy.

As stated in Section 2.8, we prepared the blood samples for the development from porcine blood. Although it is very similar to human blood, the calibration tables do not perfectly fit the practical application. A re-calibration with human blood was not conducted prior to the study since the developers had no access to human blood samples. A re-calibration at the hospital site was not possible since the procedure is technically involved and developers could not travel due to COVID-19 restrictions. Still, the achieved results during the clinical study were more than satisfying.

For system demonstration, we used a cheap camera without housing and other accessories. This camera was not designed for medical applications but rather for rapid prototyping. As such, it is relatively large (40 × 40 × 38 mm) and the main reason for the current overall sensor size of 44 × 44 × 85 mm. Additionally, the constant current supply electronic is relatively large (40 × 23 × 12 mm). Using a miniaturized camera and an integrated electronic, the overall size of the sensor can easily be reduced to less than 15 × 15 × 30 mm in total. Realizing a SoC version, the sensor including evaluation electronic could be realized in a package not larger than 25 × 25 × 30 mm.

Additionally, an approach similar to that of Hageman et al. [11] can be extended into a non-invasive measurement solution, such as the one presented by Ding et al. [12]. However, such a solution is inferior to the absorption-based approach for several reasons. Firstly, in the absorption approach, the emitter and sensor can be placed in line with the test sample. This allows for a technically less-involved setup, because all geometries can be assumed to be planar and of a constant size, without significant sacrifice of accuracy. Any additional material, such as the tube or glass slides (c.f. Section 2.5 and Section 2.7) only play a minor role when considering Beer’s Law.

In contrast, a reflectivity-based system must consider a technically involved angled arrangement because the light emitter and sensor cannot be in the same position. More importantly, the tube has a significant, non-trivial impact on the reflectance characteristics. In particular, the coloring of the tube affects the perceived color, regardless of whether a color scale is used by personnel or a computerized evaluation is performed. Lastly, reflection spectra appear to be less specific than absorption spectra.

A completely different method for estimating blood concentration in the effluent could be developed based on the photoacoustic effect. Briefly, when a medium is irradiated with a sequence of light flashes, periodic heating (and cooling) occurs. The resulting alternation of volume expansion and contraction constitutes a source of sound [21]. Using laser sources with specific wavelengths and, for example, piezoelectric acoustic receivers, it is possible to measure Hb concentration with high precision and specificity. In [22] a system for in vitro measurement of Hb and $HbO_{2}$ is described. Integrating the laser emitter into a catheter, a similar system could even allow concentration measurements directly in the bladder, which would significantly reduce the response time of the sensor (cf. Section 3). On the other hand, a rather expensive optical fiber would have to be integrated into the catheter, which is a disposable product. In [23], an interesting work is presented that aims at miniaturizing a photoacoustic sensing system. However, we assume that current solutions are still relatively large, complex and expensive and often require much more energy, compared to the sensor presented here.

Lastly, the clinical study only evaluated sensor functionality, i.e., the quality of the Hb measurement as well as bubble detection during clinical application. Further processing of sensor readings to, for example, generate alarms or treatment guidance, was neither performed nor part of the study. Sensor readings were acquired blindly and evaluated retrospectively.

## 5. Conclusions

Although further investigations are required as part of an approval study in accordance with the German as well as the international Medical Devices Act, the basic practical suitability of the sensor was demonstrated. Its non-invasive applicability allows a simple extension of the existing CBI systems and thus, a gapless monitoring of the irrigation procedure. Measuring the Hb concentration using the absorption principle (Beer’s Law) provides reliable data for the application at hand. In combination with a (mobile) communication system, monitoring data can be immediately transferred to nurse guard rooms or even mobile devices. This significantly reduces the burden on nursing staff to care for an entire ward, while still ensuring effective CBI for the benefit of patients.

## Figures and Tables

**Table 1:** Listing of hardware resources used for bubble detection and MIPI interface on the FPGA. Two different implementations of the bubble detection network are given: one that complies with the current specifications regarding frame rates as well as a maximum performance version. Please note that the standard version leaves sufficient space to realize a MIPI interface to directly connect the camera.

FPGA Utilization (c7z020-clg400-1)			
Resource	Bubble Detection Standard	Bubble Detection Performance	MIPI Interface
BRAM	15%	16%	4%
DSP	5%	5%	4%
FF	20%	26%	0%
LUT	73%	99%	6%

**Table 2:** Measurement errors with respect to the measured blood concentration. With increasing blood concentration the measurement accuracy drops due to the non-linearity of Beer’s Law. We note that these values were acquired during development and were not part of the clinical study.

Measurement Error of the Sensor					
Blood concentration	4%	5%	15%	19%	21%
Absolute error	0.11%	0.17%	0.73%	1.5%	2.5%
Relative error	2.75%	3.4%	4.87%	7.9%	11.9%

## Data Availability

Not applicable.
